# FCER1G Gene Hypomethylation in Patients with Rheumatoid Arthritis

**DOI:** 10.3390/jcm11164664

**Published:** 2022-08-09

**Authors:** Dominika Podgórska, Marek Cieśla, Bogdan Kolarz

**Affiliations:** 1 Department of Internal Diseases, Institute of Medical Sciences, College of Medical Sciences, University of Rzeszow, 35-959 Rzeszow, Poland; 2 College of Medical Sciences, University of Rzeszow, 35-959 Rzeszow, Poland

**Keywords:** epigenetic, FCER1G, immunology, rheumatoid arthritis

## Abstract

Rheumatoid arthritis (RA) is a chronic autoimmune disease that, when improperly treated, leads to disability in patients. Various factors that may cause the development and activity of RA are being considered. Epigenetic factors are also receiving increasing attention. In our study, we analyzed the association between FCER1G gene methylation and RA activity. We conducted our study in 50 RA patients and 24 controls. The patients were divided into two groups in terms of high disease activity and remission. Quantitative real-time methylation-specific PCR was used to analyze the methylation status of the investigated genes. We observed that RA patients have lower levels of methylation of the FCER1G gene compared to controls, but we did not find any difference in the methylation status of this gene between patients with high disease activity and remission. The results of this study suggest that FCER1G gene methylation may be a new potential epigenetic marker of RA that is independent of disease activity.

## 1. Introduction

Rheumatoid arthritis (RA) is a chronic autoimmune disease that affects nearly 1% of the world’s population, in which progressive joint damage and systemic symptoms occur under the influence of immune processes [1]. Untreated RA leads to progressive disability that impairs daily functioning and can even result in death.

RA can be divided into serum-positive or serum-negative forms depending on the presence or absence of serum antibodies. Classification criteria take into account the presence of rheumatoid factor (RF) and/or antibodies to citrullinated peptides/proteins (ACPA, usually determined by anti-CCP assay) [2,3,4]. ACPA antibodies show high specificity and may appear many years before the diagnosis of RA [4]. It has been proven that other antibodies also appear in RA patients, including, among others, antibodies against carbamylated proteins [5,6], antibodies against malondialdehyde-acetaldehyds adducts [7], and antibodies against peptidyl-arginine deiminase type 4 [8,9].

Some roles in the predisposition to the development of RA have been attributed to genetics. 

Past studies have led to the discovery of more than 150 loci associated with RA, but associations with human leukocyte antigen (HLA) remain the strongest [10,11,12].

Based on a meta-analysis of genome-wide association studies, it is surprising that about 80% of RA risk variants are found in non-coding DNA regions. An increasing role of the predisposition to RA risk has been attributed to changes affecting gene expression, histone modifications, chromatin conformation, or transcription factors [13,14,15]. A growing number of studies highlight the impact of epigenetic changes on the pathogenesis of RA. Epigenetics affects gene transcription, leading to heritable phenotypic changes, without altering the DNA sequence itself [16,17]. Epigenetic modifications are reversible and can be modulated by environmental factors, including diet or drugs [18,19,20].

Fc epsilon receptor Ig (FCER1G) is a gene located on chromosome 1 at position 1q23.3 that was first described in 1990. It encodes the γ chain of the Fc receptor, which is the third subunit of the high-affinity immunoglobulin E (IgE) receptor (Fcε RI). In the following years, it was observed that the Fc receptor γ chain is a common component of Fc receptors that are widely expressed in various types of immune cells.

The Fc receptor is a protein found on the surface of many different cells: neutrophils, basophils, eosinophils, platelets, macrophages, B lymphocytes, NK cells, mast cells, and dendritic cells [21]. It consists of one alpha (FCER1A) ligand binding subunit, one beta (FCER1B) signal enhancer and two gamma (FCER1G) signal transducers [22,23]. Its name comes from its binding to the Fc region of antibodies (crystallizing fragment) that are attached to infected cells or invading pathogens [21]. The binding of immunoglobulins to Fc receptors is involved in important functions of the immune system, such as phagocytosis and antibody-dependent cytotoxicity [21,24,25].

FCER1G represent a functional connection between acquired and innate immunity because they link interactions between circulating antibodies (including autoantibodies) and innate immunity cells [26,27].

FCER1G expression has been studied and described in various diseases, including squamous cell carcinoma, eczema, meningioma, leukemia, glioma, kidney disease, and even in acute myocardial infarction.

In the present study, we have investigated the effect of FCER1G gene methylation on the development of RA. To the best of our knowledge, this is the first study to examine this relationship in RA.

## 2. Materials and Methods

### Patients

A total of 74 subjects, 50 patients with RA and 24 controls, were enrolled in the study. Disease severity was estimated in the disease activity score (DAS28-ESR). From the RA group, 29 patients had high disease activity (DAS28-ESR > 5.1) and 21 patients in remission (DAS28-ESR ≤ 2.6). The diagnosis of RA was made according to the 2010 ACR/EULAR [4] or 1987 ACR [28] criteria for classification depending on the time of diagnosis. Clinical characteristics of the patients and controls are presented in Table 1 and Table 2. Variables included in the characteristics were taken from medical records. ACPAs (Anti CCP assay, DiaMetra, Segrate, Italy) and RF (EIA RF IgG, TestLine Clinical Diagnostics, Brno, Czech Republic) were determined in serum by enzyme-linked immunosorbent immunoassay and absorbance reader (Tecan Infinite M200 Pro reader and Magellan software, version 7.1, Mannedorf, Switzerland).

The Ethics Committee of the Lublin Medical University approved the study (protocol number KE-0254/7/2016), and the subjects provided their written informed consent. Blood samples were collected in ethylene diamine tetraacetic acid (EDTA) and stored at −80 °C until analysis.

## 3. Methylation Study

DNA was extracted and bisulfite converted as previously described [29]. Briefly, DNA was extracted from 200 µL of frozen whole blood and 1 µg DNA was converted using the EZ DNA Methylation Gold Kit (Zymo Research, Irvine, CA, USA) according to the manufacturer’s protocol with elution volume in 50 µL. The FCER1G target region (available from: https://epd.epfl.ch/cgi-bin/get_doc?db=hgEpdNew&format=genome&entry=FCER1G_1; accessed on 25 September 2017) was found in the Eukaryotic Promoter Database (EPD) [30]. The designed primers flanked the region from −130 to +29 bp relative to the transcription start site (TSS). Using the EPD motif tool (based on JASPAR core 2018 vertebrates), we found predictive binding sites for the transcriptional repressor CTCF (the cut-off *p*-value was 0.001) at positions −256 and −93 and +57 relative to TSS. The primers were designed using MethPrimer Software, version 1.0 [31]. Their sequences are presented in Table 3. The specificity of the primers was first tested in silico using the BiSearch tool [32]. In silico analysis predicted only one specific product in the region corresponding to the FCER1G gene. Then, the specificity of the primers was assessed by PCR with fully methylated and unmethylated DNA (EpiTect PCR Control DNA Set, Qiagen, Hilden, Germany) and was performed under the conditions corresponding to QMSP. PCR amplification products were visualized on a 2% agarose gel with the size marker.

The methylation status of the FCER1G target region was evaluated using primers complementary to the methylated sequences. The Q-MSP reaction contained 300 nM of each primer, as well as 2 µL bisulfite-treated DNA. The reaction was performed using the SG qPCR Master Mix (Eurx, Gdansk, Poland) in a total volume of 10 µL in a COBAS z480 Real-Time PCR System under the thermal cycling conditions given in the mix manual in 40 amplification cycles, except that the annealing step was carried out at 62 °C for 30 s. After each reaction, a melt curve analysis was performed. All samples were evaluated in triplicate. Q-MSP efficiency for reference and target genes was also evaluated as previously described [34]. The methylation data were analyzed using the advanced relative quantification module (LightCycler 480 SW, version 1.5.1.62 SP2–UDF v.2.0.0, Roche, Basel, Switzerland), with the maximum second derivative selected as the calculation model. The results were expressed as a fold change.

### Statistical Analysis

Depending on the distribution, assessed by the Shapiro–Wilk W test, the quantitative values were presented as mean (SD) or median [25th–75th percentile]. Differences between two independent groups were compared using the Student’s *t*-test or the Mann–Whitney U test. Kruskal–Wallis ANOVA and post hoc multiple comparison analysis was used to compare data between more than two independent groups. The relationship between two continuous variables was analyzed using Spearman’s rank correlation and presented by the value of the correlation coefficient r_s_. Qualitative parameters are given as numbers with percentage and were evaluated using contingency tables with a χ^2^ test with Yates’s correction. A *p*-value < 0.05 was considered statistically significant. The analysis was performed with STATISTICA Version 13.1 (Dell Inc., Round Rock, TX, USA, 2016).

## 4. Results

**Figure 1:**
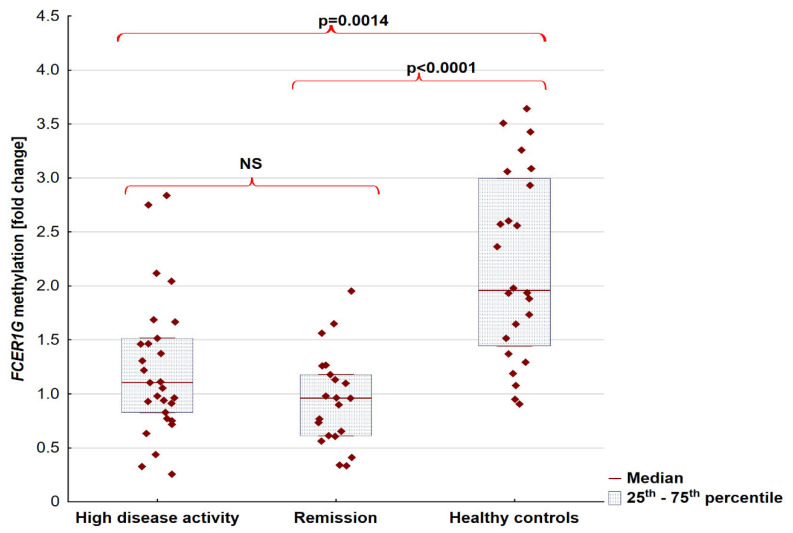
The methylation level of FCER1G in patients with rheumatoid arthritis divided into activity groups and healthy control. Abbreviations: NS, no significant.

RA patients have had a lower level of FCER1G methylation than a control group (median [25th–75th percentile]) 0.98 [0.73–1.46] vs. 1.96 [1.44–3] *p* < 0.0001). No differences in methylation status were found between RA patients in high disease activity and remission; however, both activity groups were different compared to HC (*p* = 0.0014 and *p* < 0.0001, respectively). Detailed data were presented in Figure 1.

The methylation status was only correlated with ACPA levels (−0.32) and was not correlated with other clinical variables: DAS28-ESR (r_s_ = 0.13), number of swollen joints (r_s_ = 0.21), number of painful joints (r_s_ = 0.11), ESR (r_s_ = 0.09), CRP (r_s_ = −0.1) and RF (−0.17), white blood count (r_s_ = −0.16), platelet count (r_s_ = −0.15). FCER1G methylation levels were not different (*p* = 0.21) between patients treated with methotrexate (*n* = 37) and patients without the drug (*n* = 13). In the group treated with methotrexate, there was no correlation between the dose of the drug and the level of FCER1G methylation (r_s_ = −0.14).

## 5. Discussion

In our study, we have found that RA patients had lower levels of FCER1G gene methylation compared to controls. However, we did not observe any difference in the methylation of the studied gene between the group of RA patients with high disease activity and remission, therefore, further functional experiments are necessary to explain the biological function of FCER1G and its association with the development of RA. FCER1G gene methylation may be considered as a new epigenetic marker of RA that is not related to disease activity and unrelated to common markers of inflammation, i.e., ESR or CRP. We have also found that FcRγ may play an important role in RA pathogenesis.

The pathogenesis of RA is multifactorial, with multiple immunological, genetic, epigenetic, and environmental factors playing an important role in its development.

The role of FCER1G has not been thoroughly understood and we are gaining more information about its functions over time. Studies on FCER1G have shown that it is a key molecule in signaling pathways that are widely involved in a variety of immune responses and cell types [25]. Based on mouse models, we can observe that abnormal expression of FcR can result in an uncontrolled immune response and the initiation of autoimmune disease [35]. Recent studies have shown that FcRs are important elements in several processes that, if not properly regulated, can lead to the appearance of autoreactive antibodies or autoimmune phenotypes [36,37]. Binding between the Fc of immunoglobulin and the FcR of the immune cell activates cellular effector functions through an antigen–antibody binding reaction. In a normal state of the immune system through this combination, foreign antigens are recognized and eliminated, whereas in a pathological immune response, the same combination can cause destructive inflammation, immune cell activation, phagocytosis, bursting and oxidative release of cytokines [38,39].

Activation of Fcγ receptors present on the neutrophil surface and associated with the γ chain of the Fc receptor (FcRγ) is important in mediating various cell responses, including activation of cells induced by immune complexes, removal of immune complexes, and phagocytosis of opsonized particles [35,40,41]. These processes are implicated in the pathogenesis of various autoimmune diseases, including rheumatoid arthritis [35,41,42,43].

The FCER1G gene is also involved in various biological processes, including neutrophil activation, T cell differentiation, Fc receptor-mediated signaling pathway, immunoglobulin-mediated immune response, positive regulation of interleukin-4 production, interleukin-3-mediated signaling pathway, innate immune response, positive regulation of phagocytosis, and others [44]. FCER1G may interact with members of the Dectin-2 family which was identified as a locus associated with various autoimmune disorders, including systemic lupus erythematosus and RA. It should be emphasized that there was no functional analysis related to FCER1G and RA development, therefore the matching of the FCER1G with RA development is purely theoretical and must be confirmed by functional experiments. The study conducted by Gordon et al. showed that mouse mast cells produced IL-6 when exposed to thrombin and FceRI. Thrombin is also responsible for IL-6 secretion by fibroblasts and monocytes [45], the FceRI signaling pathway has been widely studied in mast cells. Its activation is responsible for the allergic process inducing mast cells degranulation with histamine production and the synthesis of proinflammatory cytokines-TNF-α and IL-6–key molecules in the RA development. Furthermore, the release of proinflammatory cytokines was observed in dendritic cells and monocytes [46]. The study conducted by Liang et al. [47] showed that FCER1G expression is inverse to the methylation status, therefore, lower methylation is associated with higher mRNA expression. The results were obtained from peripheral blood monocytes from patients with atopic dermatitis. These data suggest that the methylation status of whole blood leukocytes may be a new epigenetic marker of gene activation and protein production. Hypomethylation of FCER1G observed in our study may be associated with higher gene expression and activation of FcRγ dependent pathway and the proinflammatory effect. Differences in methylation status were observed between patients with high disease activity and remission, but without statistical importance.

The expression of the FCER1G gene is correlated with the development of various diseases other than RA, and sometimes also affects a prognosis or response to treatment. Previous studies have shown that FCER1G is involved in the development of innate immunity and may be responsible for the development of eczema, meningioma and childhood leukemia [22,48,49]. An increased expression of FCER1G was reported in osteoarthritis cartilage compared to healthy controls [50]. A higher expression level of FCER1G has also been observed in patients with acute myocardial infarction [1]. An association between FCER1G gene expression and clear cell renal cell carcinoma (ccRCC) was also noted. The researchers noted that despite the lack of a close association between FCER1G and cancer, FCER1G expression is significantly higher in cancer cells than in normal kidney tissue. Chronic inflammation mediated by the FCER1G gene was found to be closely associated with oncogenesis [51]. The above conclusions were extended in the studies by Dong and colleagues who noted that FCER1G was more expressed in tumor tissues of ccRCC patients [52].

Our study has a few limitations. First of all, the small size of the groups means that further investigations in a larger cohort of patients are necessary to confirm our findings, especially the conclusions regarding the usefulness of FCER1G methylation as a potential marker to distinguish patients with RA and healthy controls. Moreover, subjects were enrolled in one medical center, thus an independent validation cohort is needed. The second limitation is associated with applied methodology. The methylation study was conducted based on the QMSP method, therefore results should be confirmed with a different methodology. Our finding may be considered as a basic study; further experiments are necessary to confirm the role of FCER1G in the development of RA. We compared well-established RA to healthy controls, but further studies including early RA and pre-RA patients as well as cell line models are required to better understand the role of FCER1G in disease development and pathogenesis.

In conclusion, FCER1G methylation levels may be considered as novel supportive markers in well-established RA, independent of disease activity based on DAS28 and unrelated to common laboratory markers of inflammation.

## Figures and Tables

**Table 1:** Characteristics of subjects.

Characteristics	RA Overall *n* = 50	Controls *n* = 24	*p*-Value RA Overall vs. HC
Age, years (SD)	51 (12.8)	53 (8.5)	0.45
Females	42 (84%)	17 (70.8%)	0.31
Disease duration, years	10 (3–16)	n/a	n/a
RF-positive	34 (68%)	none	n/a
ACPA-positive	46 (92%)	none	n/a
ESR, mm/h	23.5 (8–57)	15 (7–19)	0.07
DAS28	5.3 (2.11–6.12)	n/a	n/a
CRP, mg/dL	6.52 (0.51–20.76)	0.58 (0.19–1.97)	**0.003**
Number of swollen joints	3 (0–7)	n/a	n/a
Number of painful joints	4 (1–12)	n/a	n/a
VAS PGA	47.5 (8–70)	n/a	n/a
VAS PhGA	39.5 (5–60)	n/a	n/a

Data are presented as mean SD; number (%) or median [25th–75th percentile]. Abbreviations: ACPA, anti-citrullinated protein antibodies; CRP, C-reactive protein; DAS28, disease activity score; ESR, erythrocyte sedimentation rate; HC, healthy controls; RA, rheumatoid arthritis patients; RF, rheumatoid factor; VAS PhGA, visual analog scale physician global assessments; VAS PGA, visual analog scale patient global assessments. The differences between two independent groups, according to data distribution, were assessed by the Student’s *t*-test or the Mann–Whitney U test. Significant differences (*p*-value < 0.05) were bolded.

**Table 2:** Characteristics of the patients with rheumatoid arthritis.

Characteristics	RA in High Disease Activity, *n* = 29	RA in Remission, *n* = 21	*p*-Value
Age, years (SD)	54 (13.1)	47 (11.7)	0.07
Females	21 (72.4%)	21 (100%)	**0.025**
Disease duration, years	10 (3–16)	9.5 (3–16)	0.96
RF-positive	22 (75.9%)	12 (57.1%)	0.27
ACPA-positive	27 (93.1%)	19 (90.5%)	0.85
ESR, mm/h	57 (28–67)	7 (2–9)	**<0.0001**
DAS28	5.89 (5.34–6.37)	1.99 (1.58–2.31)	**<0.0001**
CRP, mg/dL	17.47 (8.58–29.1)	0.4 (0.15–1.4)	**<0.0001**
Number of swollen joints	6 (4–10)	0 (0–1)	**<0.0001**
Number of painful joints	11 (5–13)	1 (0–1)	**<0.0001**
VAS PGA	68 (55–73)	8 (3–10)	**<0.0001**
VAS PhGA	60 (49–70]	5 (2–10)	**<0.0001**
Methotrexate treatment (alone or as a concomitant drug)	22 (75.9)	15 (71.4)	0.98
Methotrexate dose, mg/week	20 (15–25)	20 (12.5–25)	0.64

Data are presented as mean SD; number (%) or median [25th–75th percentile]. Abbreviations: please refer to Table 1. The differences between two independent groups, according to data distribution, were assessed by the Student’s *t*-test or the Mann–Whitney U test. Significant differences (*p*-value < 0.05) were bolded.

**Table 3:** Characteristics of primers.

Gene	Primer Name	Sequence 5′ → 3′ ^a^	Amplicon Size [bp]	Amplicon Location with Reference to Assembly GRCh38 [Chromosome:Start:end:Strand]	Primer Complementary to Methylated/ Unmethylated Sequences
*FCER1G*	*FCER1G* methylated_sense	TGGTTTTTT**CG**GGAGT**CG**TT**C**	160	1:161215165:161215324:+	methylated sequences
	*FCER1G* methylated_antisense	CATCTTAAACTAAAAAT**CG**AC**CG**TTCT			

^a^ CpG sites in primer sequence are in bold. Abbreviations: *FCER1G*, *Fc epsilon receptor Ig gene.* To normalize DNA input after bisulfite conversion, the promoter region free of CpG sites in the beta-actin gene (*ACTB*) was amplified using the primers described previously [33]. Quantitative real-time methylation-specific PCR (Q-MSP) was used to analyze the methylation status.

## Data Availability

Data generated and analyzed during the current study are available from the corresponding author on reasonable request.

## References

[1] Mikhaylenko D.S., Nemtsova M.V., Bure I.V., Kuznetsova E.B., Alekseeva E.A., Tarasov V.V., Lukashev A.N., Beloukhova M.I., Deviatkin A.A., Zamyatnin A.A. (2020). Genetic Polymorphisms Associated with Rheumatoid Arthritis Development and Antirheumatic Therapy Response. Int. J. Mol. Sci..

[2] Padyukov L. (2022). Genetics of Rheumatoid Arthritis. Semin. Immunopathol..

[3] McInnes I.B., Schett G. (2011). The Pathogenesis of Rheumatoid Arthritis. N. Engl. J. Med..

[4] Aletaha D., Neogi T., Silman A.J., Funovits J., Felson D.T., Bingham C.O., Birnbaum N.S., Burmester G.R., Bykerk V.P., Cohen M.D. (2010). 2010 Rheumatoid Arthritis Classification Criteria: An American College of Rheumatology/European League Against Rheumatism Collaborative Initiative. Ann. Rheum. Dis..

[5] Jiang X., Trouw L.A., van Wesemael T.J., Shi J., Bengtsson C., Källberg H., Malmström V., Israelsson L., Hreggvidsdottir H., Verduijn W. (2014). Anti-CarP Antibodies in Two Large Cohorts of Patients with Rheumatoid Arthritis and Their Relationship to Genetic Risk Factors, Cigarette Smoking and Other Autoantibodies. Ann. Rheum. Dis..

[6] Volkov M., van Schie K.A., van der Woude D. (2019). Autoantibodies and B Cells: The ABC of Rheumatoid Arthritis Pathophysiology. Immunol. Rev..

[7] Strollo R., Ponchel F., Malmström V., Rizzo P., Bombardieri M., Wenham C.Y., Landy R., Perret D., Watt F., Corrigall V.M. (2013). Autoantibodies to Posttranslationally Modified Type II Collagen as Potential Biomarkers for Rheumatoid Arthritis. Arthritis Rheum..

[8] Darrah E., Giles J.T., Ols M.L., Bull H.G., Andrade F., Rosen A. (2013). Erosive Rheumatoid Arthritis Is Associated with Antibodies That Activate PAD4 by Increasing Calcium Sensitivity. Sci. Transl. Med..

[9] Kolfenbach J.R., Deane K.D., Derber L.A., O’Donnell C.I., Gilliland W.R., Edison J.D., Rosen A., Darrah E., Norris J.M., Holers V.M. (2010). Autoimmunity to Peptidyl Arginine Deiminase Type 4 Precedes Clinical Onset of Rheumatoid Arthritis. Arthritis Rheum..

[10] Okada Y., Wu D., Trynka G., Raj T., Terao C., Ikari K., Kochi Y., Ohmura K., Suzuki A., Yoshida S. (2014). Genetics of Rheumatoid Arthritis Contributes to Biology and Drug Discovery. Nature.

[11] Ha E., Bae S.-C., Kim K. (2021). Large-Scale Meta-Analysis across East Asian and European Populations Updated Genetic Architecture and Variant-Driven Biology of Rheumatoid Arthritis, Identifying 11 Novel Susceptibility Loci. Ann. Rheum. Dis..

[12] Ishigaki K., Sakaue S., Terao C., Luo Y., Sonehara K., Yamaguchi K., Amariuta T., Too C.L., Laufer V.A., Scott I.C. (2021). Trans-Ancestry Genome-Wide Association Study Identifies Novel Genetic Mechanisms in Rheumatoid Arthritis. medRxiv.

[13] Okada Y., Eyre S., Suzuki A., Kochi Y., Yamamoto K. (2019). Genetics of Rheumatoid Arthritis: 2018 Status. Ann. Rheum. Dis..

[14] Kolarz B., Podgorska D., Podgorski R. (2020). Insights of Rheumatoid Arthritis Biomarkers. Biomarkers.

[15] Hammaker D., Firestein G.S. (2018). Epigenetics of Inflammatory Arthritis. Curr. Opin. Rheumatol..

[16] Nemtsova M.V., Zaletaev D.V., Bure I.V., Mikhaylenko D.S., Kuznetsova E.B., Alekseeva E.A., Beloukhova M.I., Deviatkin A.A., Lukashev A.N., Zamyatnin A.A.J. (2019). Epigenetic Changes in the Pathogenesis of Rheumatoid Arthritis. Front. Genet..

[17] Golbabapour S., Abdulla M.A., Hajrezaei M. (2011). A Concise Review on Epigenetic Regulation: Insight into Molecular Mechanisms. Int. J. Mol. Sci..

[18] Bottini N., Firestein G.S. (2013). Epigenetics in Rheumatoid Arthritis: A Primer for Rheumatologists. Curr. Rheumatol. Rep..

[19] Glant T.T., Mikecz K., Rauch T.A. (2014). Epigenetics in the Pathogenesis of Rheumatoid Arthritis. BMC Med..

[20] Klein K., Gay S. (2013). Epigenetic Modifications in Rheumatoid Arthritis, a Review. Curr. Opin. Pharmacol..

[21] Anderson R. (2003). Manipulation of Cell Surface Macromolecules by Flaviviruses. Advances in Virus Research.

[22] Mahachie John J.M., Baurecht H., Rodríguez E., Naumann A., Wagenpfeil S., Klopp N., Mempel M., Novak N., Bieber T., Wichmann H.-E. (2010). Analysis of the High Affinity IgE Receptor Genes Reveals Epistatic Effects of FCER1A Variants on Eczema Risk. Allergy.

[23] Kraft S., Kinet J.-P. (2007). New Developments in FcεRI Regulation, Function and Inhibition. Nat. Rev. Immunol..

[24] Küster H., Thompson H., Kinet J.P. (1990). Characterization and Expression of the Gene for the Human Fc Receptor Gamma Subunit. Definition of a New Gene Family. J. Biol. Chem..

[25] Fu L., Cheng Z., Dong F., Quan L., Cui L., Liu Y., Zeng T., Huang W., Chen J., Pang Y. (2020). Enhanced Expression of FCER1G Predicts Positive Prognosis in Multiple Myeloma. J. Cancer.

[26] Andreu P., Johansson M., Affara N.I., Pucci F., Tan T., Junankar S., Korets L., Lam J., Tawfik D., DeNardo D.G. (2010). FcRγ Activation Regulates Inflammation-Associated Squamous Carcinogenesis. Cancer Cell.

[27] Nimmerjahn F., Ravetch J.V. (2008). Fcγ Receptors as Regulators of Immune Responses. Nat. Rev. Immunol..

[28] Arnett F.C., Edworthy S.M., Bloch D.A., McShane D.J., Fries J.F., Cooper N.S., Healey L.A., Kaplan S.R., Liang M.H., Luthra H.S. (1988). The American Rheumatism Association 1987 Revised Criteria for the Classification of Rheumatoid Arthritis. Arthritis Rheum..

[29] Podgórska D., Cieśla M., Majdan M., Podgórski R., Kolarz B. (2021). The Relationship of ADAMTSL2 and LRPAP1 Gene Methylation Level with Rheumatoid Arthritis Activity. Clin. Exp. Rheumatol..

[30] Dreos R., Ambrosini G., Périer R.C., Bucher P. (2015). The Eukaryotic Promoter Database: Expansion of EPDnew and New Promoter Analysis Tools. Nucleic Acids Res..

[31] Li L.-C., Dahiya R. (2002). MethPrimer: Designing Primers for Methylation PCRs. Bioinformatics.

[32] Tusnády G.E., Simon I., Váradi A., Arányi T. (2005). BiSearch: Primer-Design and Search Tool for PCR on Bisulfite-Treated Genomes. Nucleic Acids Res..

[33] Cieśla M., Kolarz B., Majdan M., Darmochwał-Kolarz D. (2019). IRF5 Promoter Methylation as a New Potential Marker of Rheumatoid Arthritis. Pol. Arch. Intern. Med..

[34] Livak K.J., Schmittgen T.D. (2001). Analysis of Relative Gene Expression Data Using Real-Time Quantitative PCR and the 2(-Delta Delta C(T)) Method. Methods.

[35] Nimmerjahn F. (2006). Activating and Inhibitory FcγRs in Autoimmune Disorders. Springer Semin. Immun..

[36] Takai T. (2002). Roles of Fc Receptors in Autoimmunity. Nat. Rev. Immunol..

[37] Ravetch J.V., Kinet J.P. (1991). Fc Receptors. Annu. Rev. Immunol..

[38] Liu S., Wang C., Yang H., Zhu T., Jiang H., Chen J. (2020). Weighted Gene Co-Expression Network Analysis Identifies FCER1G as a Key Gene Associated with Diabetic Kidney Disease. Ann. Transl. Med..

[39] Brandsma A.M., Hogarth P.M., Nimmerjahn F., Leusen J.H.W. (2016). Clarifying the Confusion between Cytokine and Fc Receptor “Common Gamma Chain”. Immunity.

[40] Bruhns P., Jönsson F. (2015). Mouse and Human FcR Effector Functions. Immunol. Rev..

[41] Futosi K., Mócsai A. (2016). Tyrosine Kinase Signaling Pathways in Neutrophils. Immunol. Rev..

[42] Ludwig R.J., Vanhoorelbeke K., Leypoldt F., Kaya Z., Bieber K., McLachlan S.M., Komorowski L., Luo J., Cabral-Marques O., Hammers C.M. (2017). Mechanisms of Autoantibody-Induced Pathology. Front. Immunol..

[43] Németh T., Futosi K., Szabó M., Aradi P., Saito T., Mócsai A., Jakus Z. (2019). Importance of Fc Receptor γ-Chain ITAM Tyrosines in Neutrophil Activation and in Vivo Autoimmune Arthritis. Front. Immunol..

[44] Cunningham F., Allen J.E., Allen J., Alvarez-Jarreta J., Amode M.R., Armean I.M., Austine-Orimoloye O., Azov A.G., Barnes I., Bennett R. (2022). Ensembl 2022. Nucleic Acids Res..

[45] Gordon J.R., Zhang X., Stevenson K., Cosford K. (2000). Thrombin Induces IL-6 but Not TNFalpha Secretion by Mouse Mast Cells: Threshold-Level Thrombin Receptor and Very Low Level FcepsilonRI Signaling Synergistically Enhance IL-6 Secretion. Cell Immunol..

[46] Shin J.-S., Greer A.M. (2015). The Role of FcεRI Expressed in Dendritic Cells and Monocytes. Cell. Mol. Life Sci..

[47] Liang Y., Wang P., Zhao M., Liang G., Yin H., Zhang G., Wen H., Lu Q. (2012). Demethylation of the FCER1G Promoter Leads to FcεRI Overexpression on Monocytes of Patients with Atopic Dermatitis. Allergy.

[48] Rajaraman P., Brenner A.V., Neta G., Pfeiffer R., Wang S.S., Yeager M., Thomas G., Fine H.A., Linet M.S., Rothman N. (2010). Risk of Meningioma and Common Variation in Genes Related to Innate Immunity. Cancer Epidemiol. Biomark. Prev..

[49] Han S., Lan Q., Park A.K., Lee K.-M., Park S.K., Ahn H.S., Shin H.Y., Kang H.J., Koo H.H., Seo J.J. (2010). Polymorphisms in Innate Immunity Genes and Risk of Childhood Leukemia. Hum. Immunol..

[50] Karlsson C., Dehne T., Lindahl A., Brittberg M., Pruss A., Sittinger M., Ringe J. (2010). Genome-Wide Expression Profiling Reveals New Candidate Genes Associated with Osteoarthritis. Osteoarthr. Cartil..

[51] Chen L., Yuan L., Wang Y., Wang G., Zhu Y., Cao R., Qian G., Xie C., Liu X., Xiao Y. (2017). Co-Expression Network Analysis Identified FCER1G in Association with Progression and Prognosis in Human Clear Cell Renal Cell Carcinoma. Int. J. Biol. Sci..

[52] Dong K., Chen W., Pan X., Wang H., Sun Y., Qian C., Chen W., Wang C., Yang F., Cui X. (2022). FCER1G Positively Relates to Macrophage Infiltration in Clear Cell Renal Cell Carcinoma and Contributes to Unfavorable Prognosis by Regulating Tumor Immunity. BMC Cancer.

